# Peritoneal dialysis in rural Australia

**DOI:** 10.1186/1471-2369-14-278

**Published:** 2013-12-20

**Authors:** Nicholas A Gray, Blair S Grace, Stephen P McDonald

**Affiliations:** 1 Department of Renal Medicine, Nambour General Hospital, Nambour, Queensland, Australia; 2 The University of Queensland, Sunshine Coast Clinical School, Nambour General Hospital, Nambour, Queensland, Australia; 3 Australia and New Zealand Dialysis and Transplant Registry, Adelaide, South Australia, Australia; 4 Discipline of Medicine, University of Adelaide, Adelaide, South Australia, Australia; 5 Department of Renal Medicine, Central and North Adelaide Renal Services, Royal Adelaide Hospital, Adelaide, South Australia, Australia

**Keywords:** ANZDATA, Australia, Dialysis, Mortality, Outcomes, Peritoneal dialysis, Remoteness, Rural

## Abstract

**Background:**

Australians living in rural areas have lower incidence rates of renal replacement therapy and poorer dialysis survival compared with urban dwellers. This study compares peritoneal dialysis (PD) patient characteristics and outcomes in rural and urban Australia.

**Methods:**

Non-indigenous Australian adults who commenced chronic dialysis between 1 January 2000 and 31 December 2010 according to the Australia and New Zealand Dialysis and Transplant Registry (ANZDATA) were investigated. Each patient’s residence was classified according to the Australian Bureau of Statistics remote area index as major city (MC), inner regional (IR), outer regional (OR), or remote/very remote (REM).

**Results:**

A total of 7657 patients underwent PD treatment during the study period. Patient distribution was 69.0% MC, 19.6% IR, 9.5% OR, and 1.8% REM. PD uptake increased with increasing remoteness. Compared with MC, sub-hazard ratios [95% confidence intervals] for commencing PD were 1.70 [1.61-1.79] IR, 2.01 [1.87-2.16] OR, and 2.60 [2.21-3.06] REM. During the first 6 months of PD, technique failure was less likely outside MC (sub-hazard ratio 0.47 [95% CI: 0.35-0.62], P < 0.001), but no difference was seen after 6 months (sub-hazard ratio 1.05 [95% CI: 0.84-1.32], P = 0.6). Technique failure due to technical (sub-hazard ratio 0.57 [95% CI: 0.38-0.84], P = 0.005) and non-medical causes (sub-hazard ratio 0.52 [95% CI: 0.31-0.87], P = 0.01) was less likely outside MC. Time to first peritonitis episode was not associated with remoteness (P = 0.8). Patient survival while on PD or within 90 days of stopping PD did not differ by region (P = 0.2).

**Conclusions:**

PD uptake increases with increasing remoteness. In rural areas, PD technique failure is less likely during the first 6 months and time to first peritonitis is comparable to urban areas. Mortality while on PD does not differ by region. PD is therefore a good dialysis modality choice for rural patients in Australia.

## Background

Among non-indigenous Australians, the incidence of renal replacement therapy and survival on dialysis are lower in rural compared with urban areas [1]. Peritoneal dialysis (PD) prevalence in Australia has fallen from 27% in 2000 [2] to 21% in 2009 [3]. However, the uptake of PD among dialysis patients living in rural areas of Australia [1] and USA [4,5], and Canadians living more than 50km from the treating nephrologist [6], has been shown to be higher than urban dwellers.

The impact of rural residence on PD outcomes is less well understood, particularly outside North America. Canadian patients living more than 50 km from their treating nephrologist were less likely to suffer technique failure and transfer to haemodialysis (HD), but suffered increased mortality [6]. A smaller study from Ontario, Canada did not show a difference in technique failure or mortality with PD in rural areas [7]. In the USA, rural PD patients have a higher mortality risk than those in urban areas [5]. In Australia, technique failure, peritonitis and mortality have been shown to be higher among remote living indigenous PD patients compared with urban dwellers [8]. Time to the first episode of peritonitis among Australian PD patients living more than 100km from the treating centre is shorter than those living within 100km [9]. Different management practices for peritonitis for patients living distant to the treating centre have also been reported [9].

Many studies have examined distance from the treating centre rather than rural residence per se. However, rural residence has been shown to be directly linked with multiple poor health outcomes [10]. This paper describes non-indigenous PD patient characteristics, complications, and outcomes in rural Australia.

## Methods

The Australia and New Zealand Dialysis and Transplant Registry (ANZDATA) collects observational data on all patients receiving chronic renal replacement therapy in Australia. All data are collected and submitted to ANZDATA by the treating nephrologist or renal health team at each local site. This study included all non-indigenous patients aged > =18 years registered with ANZDATA, who commenced renal replacement therapy between 1 January 2000 and 31 December 2010, and underwent PD at some stage during this period in Australia. Indigenous patients (those who self-identify as Australian Aborigines or Torres Strait Islanders when asked their racial origin) undertaking PD were excluded from this analysis because work in this area has already been completed [8,11].

The Australian Bureau of Statistics used 2006 Census data to produce the Australian Standard Geographical Classification of remoteness areas [12]. This classifies all statistical local areas according to a remote area index which is determined by measuring the road distance from a statistical local area to five classes of service centre. There are six remote area index classifications: major city (MC), inner regional, outer regional, remote, very remote and migratory. Urban areas include the MC category, while rural areas include regional, remote and very remote Australia. Where a postcode contained statistical local areas from two or more remote area index classifications, the postcode was allocated the remote area index that had the greatest population. Australian patients without postcode data recorded at commencement of renal replacement therapy were excluded. Patient numbers in the remote and very remote areas were small so these groups were combined into a single remote category.

Time to first use of PD was analysed for all patients who started renal replacement therapy by either continuous ambulatory PD (CAPD) or automated PD (APD). The use of icodextrin among PD patients was recorded during annual surveys in 2007 – 2010. We analysed use of icodextrin at the survey closest to 1 year after commencing renal replacement therapy for patients who commenced PD in this period.

Technique failure was defined as any change of modality from PD to HD that lasted more than 30 days. Reasons for technique failure were only recorded after 2006 and were coded as infectious (related to peritonitis, tunnel or exit site infection), technical (dialysate leak, hydrothorax, scrotal oedema, catheter difficulties, hernia, pain, surgery, adhesion), dialysis related (ultrafiltration or solute clearance), non-medical (patient choice for personal reasons), transplantation, death, and miscellaneous.

The date of first episode of peritonitis was recorded from 2000, but more detailed data related to peritonitis (type of organism, treating antibiotics, outcomes of treatment) was only routinely collected after October 2003. Peritonitis outcomes were classified as resolution of peritonitis with continuation of PD, removal of Tenckhoff catheter, permanent transfer to HD, and death within 90 days.

Patient death on PD was defined two ways: death during PD treatment; and death while on PD or within 90 days of transferring to HD. Patient death following transplantation but within 90 days of ceasing PD was considered a transplant related death. Causes of death were categorised into: cardiovascular causes (cardiac complications, ischaemia, infarction, aneurysms, haemorrhage), infectious, non-medical (suicide, withdrawal from dialysis for any reason, accidental death), malignant or miscellaneous. Patient survival was analysed using an “as treated analysis.”

### Statistics

All analyses were adjusted for the following factors at commencement of renal replacement therapy: age category (18–44, 45–54, 55–64, 65+ years), body mass index category (< 18.49, 18.5-24.9, 25–29.9, 30+ kg/m^2^), smoking status, comorbidities (diabetes, chronic lung disease, coronary artery disease, peripheral vascular disease, cerebrovascular disease), primary kidney disease (glomerulonephritis, diabetes, hypertension, polycystic, reflux or others), late referral (commencing renal replacement therapy within 3 months of referral to nephrology care), gender, race (Caucasian, Asian or other), and size of initial treating centre. The size of the initial treating centre was divided based on the number of incident patients from 2000–2010; small (1–49 patients), medium (50–199 patients), and large (200+ patients).

Uptake of all forms of PD, as well as patient and technique failure, and time to first episode of peritonitis were all analysed using competing risk regressions, using the methods of Fine and Gray [13]. The assumption of constant proportional sub-hazards was checked by plotting Shoenfeld-like residuals and by investigating a remoteness: time interaction term within the model. For analyses of PD uptake from time of commencing renal replacement therapy, death and transplantation were competing risks. Death, transplantation and APD were competing risks for CAPD. Death, transplantation and CAPD were competing risks for APD.

All cause technique failure from the time of commencing PD was analysed with transplantation as a competing risk. Because the hazard ratio varied over time, this analysis was stratified. Technique failure within the first 6 months was examined separately to subsequent failure. Technique failure due to a specific cause was analysed with other causes as competing risks. Patients were encoded as either MC or other when small numbers of cases were present (individual causes of PD technique failure and cause of death). Time to first peritonitis episode analyses included death and transplantation as competing risks. The incidence of peritonitis was analysed using Poisson regression, with total time on PD per patient as an offset.

The outcomes of each case of peritonitis were investigated using mixed-effects logistic regression, with infection number nested within patient as random effects. Separate models investigated the proportion of peritonitis cases that resulted in a permanent change to HD, removal of the peritoneal catheter, or death within 90 days. The use of icodextrin at the survey closest to one year after commencing renal replacement therapy was analysed using logistic regression. Specific causes of death were also compared between remoteness areas using logistic regression.

Results are presented as either sub-hazard ratios (analogous to hazard ratios from Cox regressions) from competing risk survival models, or odds ratio from logistic regressions, with 95% confidence intervals [95% CI].

All analysis was carried out using Stata IC 12.1 (StataCorp, College Station, TX, USA). This study was approved by the Prince Charles Hospital human research ethics committee.

## Results

**Figure 1:**
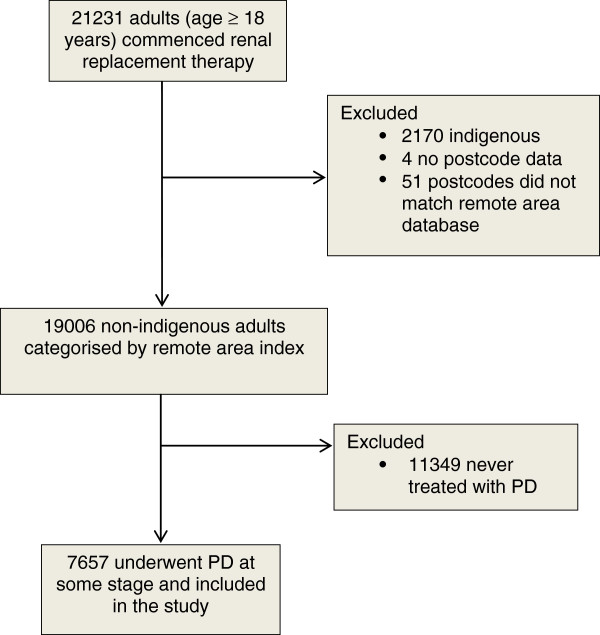
**Flowchart of patients included in the study.** PD = peritoneal dialysis.

Figure 1 shows a flow chart of the study population. PD patient distribution was 69.0% MC, 19.6% inner regional, 9.5% outer regional, and 1.8% remote. Patient characteristics at commencement of renal replacement therapy are shown in Table 1. Diabetic kidney disease was more common in MC while current smoking, chronic lung disease, obesity and Caucasian race were more common among patients from rural areas.

**Table 1:** Characteristics of adult non-indigenous Australian patients who commenced renal replacement therapy (2000–2010) and underwent PD at some stage

**Factor**	**Major city**	**Inner regional**	**Outer regional**	**Remote**	**p-value**
**N**	**5285**	**1503**	**731**	**138**	
Age, median (IQR)	63 (50, 72)	64 (52, 71)	64 (52, 72)	64 (51, 72)	0.64
Male	57.8%	57.7%	59.2%	60.9%	0.80
Body mass index (kg/m^2^)					<0.001
Underweight (<18.5)	4.2%	2.5%	3.4%	1.4%	
Normal (18.5–24.9)	40.3%	38.9%	37.2%	43.5%	
Overweight (25–29.9)	33.9%	34.0%	36.6%	22.5%	
Obese (> = 30)	21.6%	24.5%	22.7%	32.6%	
Chronic lung disease	13.7%	15.9%	14.1%	21.0%	0.02
Coronary artery disease	34.7%	34.9%	32.1%	33.3%	0.60
Peripheral vascular disease	20.0%	20.7%	17.8%	22.5%	0.30
Cerebrovascular disease	14.3%	15.6%	13.3%	16.7%	0.40
Diabetes	40.2%	32.5%	33.9%	33.3%	<0.001
Primary renal disease					<0.001
Glomerulonephritis	1422 (26.9%)	430 (28.6%)	184 (25.2%)	34 (24.6%)	
Diabetes	1633 (30.9%)	341 (22.7%)	176 (24.1%)	38 (27.5%)	
Hypertension	523 (9.9%)	149 (9.9%)	78 (10.7%)	25 (18.1%)	
Polycystic	313 (5.9%)	103 (6.9%)	47 (6.4%)	8 (5.8%)	
Reflux	243 (4.6%)	75 (5.0%)	26 (3.6%)	6 (4.3%)	
Other	1151 (21.8%)	405 (26.9%)	220 (30.1%)	27 (19.6%)	
Late referral	21.2%	23.4%	24.2%	25.4%	0.10
Current smoking	10.6%	12.0%	13.7%	14.5%	0.03
Race					<0.001
Caucasian	77.4%	97.0%	94.7%	94.2%	
Asian	12.9%	1.7%	3.1%	2.2%	
Other	9.7%	1.3%	2.2%	3.6%	
Creatinine (umol/L) at entry, median (IQR)	631 (490, 827)	635 (490, 813)	673 (536, 855)	729 (540, 910)	<0.001
PD at commencement of dialysis	3446 (65.2%)	1002 (66.7%)	433 (59.2%)	82 (59.4%)	0.002
PD facility size (incident patients in study period)					<0.001
1–49 patients	139 (2.6%)	86 (5.7%)	53 (7.3%)	26 (18.8%)	
50–199 patients	314 (5.9%)	488 (32.5%)	266 (36.4%)	32 (23.2%)	
200+ patients	4829 (91.4%)	929 (61.8%)	412 (56.4%)	80 (58.0%)	
Peritonitis cases per year (Poisson mean and 95%CI)	0.55 (0.53–0.56)	0.55 (0.51–0.58)	0.59 (0.54–0.64)	0.71 (0.59–0.84)	0.014
Time per patient spent on PD in months, median (IQR)	16.4 (7.1–29.3)	15.8 (7.7–29.3)	17.0 (7.6–27.8)	17.0 (7.8–31.7)	0.81

*IQR* interquartile range, late referral = commenced dialysis < 3 months from first referral to a nephrologist, PD facility size was categorised by the number of patients commencing PD during the study period, *CI* confidence interval.

**Figure 2:**
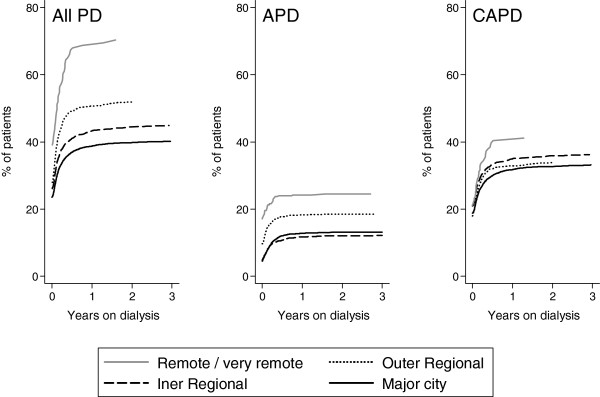
Uptake of all PD, CAPD and APD over time, by remoteness categories.

The uptake of PD increased with increasing remoteness (Figure 2). However, among patients who ever used PD, patients from outer regional and remote areas were less likely to commence renal replacement therapy with PD (Table 1). Compared with MC, sub-hazard ratios [95% confidence intervals] for uptake of PD after adjustment for age, gender, body mass index, smoking, comorbidities, late referral, race, and centre size were 1.70 [1.61-1.79] inner regional, 2.01 [1.87-2.16] outer regional, and 2.60 [2.21-3.06] remote. This was largely due to increasing uptake of APD, although CAPD also increased with remoteness. Use of icodextrin did not vary significantly with remoteness (P = 0.9).

**Figure 3:**
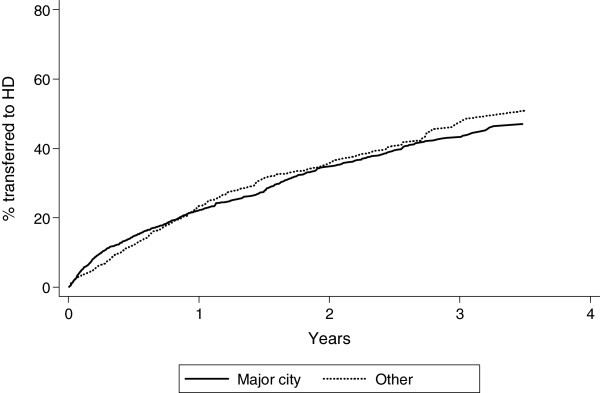
**Cumulative incidence of technique failure, with transplantation and death as competing risks.** Inner regional, outer regional and remote areas have been combined to a single “other” group due to small numbers.

Technique failure rates in rural areas were low so inner regional, outer regional and remote patients were grouped. Overall technique failure was less likely during the first 6 months of PD for this rural group compared with MC after adjusting for other variables, but technique failure was not associated with remoteness after 6 months of PD (Figure 3). Patients living outside major cities were less likely to be transferred to HD due to technical or non-medical causes (Table 2).

**Table 2:** Competing risk sub-hazard ratios [95% confidence intervals] for technique failure, by reason for failure

	**Major city**	All other regions*
All cause – first 6 months	1 (reference)	0.47 [0.35–0.62], P < 0.001
All cause > 6 months	1 (reference)	1.05 [0.84–1.32], P = 0.6
Infection	1 (reference)	1.15 [0.80–1.67], P = 0.5
Dialysis	1 (reference)	1.05 [0.73–1.52], P = 0.8
Technical	1 (reference)	0.57 [0.38–0.84], P = 0.005
Non–medical	1 (reference)	0.52 [0.31–0.87], P = 0.01
Transplantation	1 (reference)	1.12 [0.82–1.53], P = 0.5

*Inner regional, outer regional and remote were grouped due to small numbers.

Each cause of failure has all other causes and death as competing risks. Data for reason for technique failure was only available after 2006.

There were 5159 cases of peritonitis over 9328 person-years at risk, giving a rate of 0.55 cases per year. Time to first peritonitis episode did not vary with remoteness (P = 0.8), but total peritonitis cases per year did (Table 1). Compared to small centres, medium-sized centres (50 – 199 incident patients) and large centres (200+ incident patients) had lower rates of peritonitis (sub-hazard ratio [95% CI] for time to first peritonitis 0.64 [0.50-0.81], P < 0.001; and 0.60 [0.45-0.74], P < 0.001 respectively). Patients living outside major cities were more likely to suffer culture negative or methicillin sensitive *Staphylococcus aureus* peritonitis (Table 3). Remote patients were less likely to transfer permanently to haemodialysis after an episode of peritonitis (Table 4).

**Table 3:** Distribution of agents causing first episode of peritonitis, by remoteness area

	**Major city**	**Inner regional**	**Outer regional**	**Remote**	**p-value**
Culture negative	257 (14.5%)	103 (19.9%)	42 (16.3%)	9 (16.4%)	0.03
Coagulase negative *Staphylococcus aureus*	432 (24.4%)	110 (21.3%)	50 (19.4%)	9 (16.4%)	0.4
Methicillin resistant *Staphylococcus aureus*	26 (1.5%)	13 (2.5%)	5 (1.9%)	3 (5.5%)	0.4
Methicillin sensitive *Staphylococcus aureus*	138 (7.8%)	38 (7.4%)	33 (12.8%)	5 (9.1%)	0.01
Other gram positive	307 (17.3%)	77 (14.9%)	37 (14.3%)	7 (12.7%)	0.9
Gram negative	481 (27.1%)	129 (25.0%)	69 (26.7%)	15 (27.3%)	0.8
Anaerobes	4 (0.2%)	4 (0.8%)	0 (0.0%)	0 (0.0%)	0.04
Fungi	45 (2.5%)	14 (2.7%)	10 (3.9%)	1 (1.8%)	0.6
Mycobacteria	7 (0.4%)	2 (0.4%)	2 (0.8%)	0 (0.0%)	0.6
Other	73 (4.1%)	25 (4.8%)	10 (3.9%)	5 (9.1%)	0.09
No culture taken	3 (0.2%)	2 (0.4%)	0 (0.0%)	1 (1.8%)	0.6

Percentages are for each column and can be > 100% because of multiple organisms cultured for some patients.

**Table 4:** Peritonitis outcomes

	**Major city**	**Inner regional**	**Outer regional**	**Remote**
Transfer to haemodialysis	1 (reference)	0.89 [0.73–1.10] P = 0.3	0.91 [0.70–1.18] P = 0.5	0.49 [0.28–0.88] P = 0.02
Catheter removal	1 (reference)	0.93 [0.76–1.14] P = 0.5	0.96 [0.75–1.24] P = 0.8	0.75 [0.45–1.22] P = 0.2
Death within 90 days	1 (reference)	0.94 [0.58–1.53] P = 0.8	1.13 [0.60–2.12] P = 0.7	1.08 [0.32–3. 61] P = 0.9

Data presented are odds ratios [95% confidence intervals] and P values, produced from mixed–effects logistic regression. Models were adjusted for age, body mass index category, smoking status, comorbidities, primary kidney disease, late referral, gender, race, and size of treating centre, with infection number nested within patient as random effects.

Patient survival while on PD or within 90 days of stopping PD did not differ significantly by region overall (P = 0.2). Cause of death between major cities and grouped rural areas did not differ for infectious (P = 0.7), non-medical causes including withdrawal from dialysis for any reason, accident, or suicide (P = 0.1), or miscellaneous causes (P = 0.8). There was a trend towards increased cardiovascular (sub-hazard ratio 1.13 [95% CI: 0.98-1.29], P = 0.09) and malignant (sub-hazard ratio 1.49 [95% CI: 0.99-2.27], P = 0.06) deaths in rural areas.

## Discussion

This study has shown that among non-indigenous Australians, the uptake of PD increases with increasing remoteness; time to first peritonitis in rural areas is comparable with MC; PD technique failure rates in the first 6 months are lower in rural areas due to less technical and non-medical causes; and overall death rates do not vary between regions. There is a suggestion that more cardiovascular and malignant deaths occur in rural areas.

Incidence rates of renal replacement therapy among non-indigenous people have previously been shown to be lower in rural Australia [1]. PD is suited to many patients living in remote areas, where regular travel to HD units is not practical. In addition, PD does not need large quantities of clean water and/or reliable power which may limit home haemodialysis as an option for some rural patients. It is therefore not surprising that PD uptake increases with remoteness. Our data confirms findings from other studies [4-6].

Importantly, our data showed no difference in mortality among PD patients across all regions. This is reassuring for non-indigenous rural patients that they can safely undertake PD and not be disadvantaged. Given the overall increased mortality risk for dialysis patients in rural Australia [1], these findings suggest PD is a preferred modality. While these findings are supported by a Canadian cohort of incident PD patients [7], in the USA PD patients had higher mortality in micropolitan and rural areas [5]. The prevalence of PD is much lower in USA and there are likely many other country specific and patient selection factors that may explain the difference. A limitation of our study is that we did not measure distance to the treating centre which has been associated with increased PD patient mortality [6] or peritonitis risk [9]. Furthermore, our data do not apply to indigenous patients in rural areas of Australia who have been shown to have higher mortality rates than non-indigenous, possibly due to a shorter time to first peritonitis and that 79% of indigenous in rural Australia live in remote areas, whereas most non-indigenous in rural Australia are in regional areas [8].

Technique failure rates in rural areas were lower during the first 6 months, similar to Canadian data [6]. In our cohort this was mainly due to fewer technical and non-medical reasons for failure. It is understandable that if a rural patient has a significant distance to travel for HD there may be greater incentive to persist with PD rather than abandon for personal reasons. Furthermore, travel time has been associated with increased mortality on haemodialysis [14], so it is sensible to continue with PD when possible. The reason for fewer technical complications causing technique failure in rural PD patients is uncertain, but may relate to greater persistence in rural areas to resolve technical problems and continue PD. Importantly, PD failure for dialysis reasons such as inadequate clearances or ultrafiltration were similar between regions, suggesting that dialysis adequacy was not compromised. Furthermore, although more people underwent PD, the total duration of PD treatment in months did not differ by region. PD technique failure after 6 months did not differ by region, perhaps due to the greater uptake of PD among rural patients resulting in people less suited to self care commencing PD than in urban areas.

Time to first episode of peritonitis and peritonitis as a cause for technique failure were not different by remoteness area. We did find a difference in overall peritonitis rates by region, with higher rates in remote areas in particular. However, peritonitis data submitted to ANZDATA for second and subsequent episodes may not be as accurate or complete as for the first episode. Previous work has shown an increased rate of peritonitis and shorter time to first peritonitis for patients living >100km from the treating centre in Australia [9]. It seems that distance from the treating centre and possibly remote residence is therefore associated with increased peritonitis rates in Australia. This may reflect difficulties with home visits with increased distance and suggests a possible role for telemedicine.

Our study confirms previous findings [9] that there is an increased rate of *Staphylococcus aureus* peritonitis in rural areas, especially in outer regional and remote Australia. The causes for this finding are uncertain but may be affected by higher *Staphylococcus aureus* colonisation rates and possibly inadequate decolonisation procedures in remote areas. Decolonisation with topical mupirocin has been associated with a 70% reduction in *Staphylococcus aureus* peritonitis rates [15].

Our data show that while PD uptake is more common in rural areas, those living in outer regional and remote Australia are less likely to commence dialysis with PD than in major cities. This finding is different to Canadian [6] and American [5] data which shows people in remote areas are more likely to commence dialysis with PD than city dwellers. However, in USA the uptake of PD at commencement of dialysis was less than 10% of incident patients, much lower than Australia. The Canadian study was different to ours because it examined patients commencing dialysis in an earlier time period (1990–2000), examined distance from the treating nephrologist rather than rural residence, and included indigenous patients. The lower dialysis initiation with PD in outer regional and remote areas was not explained by a difference in late referral to a nephrologist by remoteness area. However it remains possible that people in these areas did not seek nephrology care until late in the course of their kidney disease, perhaps due to the smaller medical [16] and nephrology [17] workforces compared with urban areas.

Our study has several limitations. Postcode data at commencement of renal replacement therapy was used and we do not know how many or how often patients relocate to access health care for kidney disease prior to commencing dialysis. Socio-economic status was not examined in this analysis. Previous work has demonstrated increased PD technique failure with lower neighbourhood education level [7]. Furthermore, lower socioeconomic status has been associated with increased PD patient peritonitis and mortality in China [18] and peritonitis associated hospitalisation and death in Australia [19]. The method used to classify a postcode by remote area index relied on data from statistical local areas and some postcodes had several different remote area index classifications of statistical local areas. Our classification of each postcode may thus have created a bias, although to minimise this we used the most populous remote area index allocation for each postcode. There is no data on patients who were managed conservatively and never commenced dialysis, although treatment rates have been shown similar by remoteness [20]. The data is observational and there are many variables such as exit-site infection, local protocols, use of telehealth, and local available expertise which are unavailable for this analysis. Lastly, the data collected by ANZDATA is submitted voluntarily and has only been subjected to a small audit [21], although all units in Australia and New Zealand participate and assert that reporting is complete.

## Conclusion

This study has shown an increased uptake of PD with increasing remoteness in Australia. PD technique failure rates are lower in rural areas while peritonitis rates and mortality do not vary by region. PD therefore appears to be a good treatment choice for patients living in rural Australia. Efforts to maintain and improve quality care in rural areas such as adherence to guidelines, use of outreach clinics and telehealth may further enhance the health of rural patients.

## Abbreviations

PD: Peritoneal dialysis; ANZDATA: Australia and New Zealand Dialysis and Transplant Registry; MC: Major city; IR: Inner regional; OR: Outer regional; REM: Remote/very remote; CI: Confidence intervals; HD: Haemodialysis; CAPD: Continuous ambulatory peritoneal dialysis; APD: Automated peritoneal dialysis.

## Competing interests

The authors declare that they have no competing interests.

## Authors’ contributions

NG conceived the study, participated in its design and coordination and drafted the manuscript. BG participated in study design, performed the statistical analysis and participated in drafting the manuscript. SM participated in study design and statistical analysis. All authors read and approved the final manuscript.

## Pre-publication history

The pre-publication history for this paper can be accessed here:

http://www.biomedcentral.com/1471-2369/14/278/prepub
